# The *Salmonella* Effector SpvD Is a Cysteine Hydrolase with a Serovar-specific Polymorphism Influencing Catalytic Activity, Suppression of Immune Responses, and Bacterial Virulence*[Fn FN2]

**DOI:** 10.1074/jbc.M116.752782

**Published:** 2016-10-27

**Authors:** Grzegorz J. Grabe, Yue Zhang, Michal Przydacz, Nathalie Rolhion, Yi Yang, Jonathan N. Pruneda, David Komander, David W. Holden, Stephen A. Hare

**Affiliations:** From the ‡ Section of Microbiology and; § Department of Life Sciences, MRC Centre for Molecular Bacteriology and Infection, Imperial College London, London SW7 2AZ, United Kingdom and; the ¶ Division of Protein and Nucleic Acid Chemistry, MRC Laboratory of Molecular Biology, Cambridge CB2 OQH, United Kingdom

**Keywords:** bacterial pathogenesis, crystal structure, cysteine protease, Salmonella enterica, structure-function, effector

## Abstract

Many bacterial pathogens secrete virulence (effector) proteins that interfere with immune signaling in their host. SpvD is a *Salmonella enterica* effector protein that we previously demonstrated to negatively regulate the NF-κB signaling pathway and promote virulence of *S. enterica* serovar Typhimurium in mice. To shed light on the mechanistic basis for these observations, we determined the crystal structure of SpvD and show that it adopts a papain-like fold with a characteristic cysteine-histidine-aspartate catalytic triad comprising Cys-73, His-162, and Asp-182. SpvD possessed an *in vitro* deconjugative activity on aminoluciferin-linked peptide and protein substrates *in vitro*. A C73A mutation abolished SpvD activity, demonstrating that an intact catalytic triad is required for its function. Taken together, these results strongly suggest that SpvD is a cysteine protease. The amino acid sequence of SpvD is highly conserved across different *S. enterica* serovars, but residue 161, located close to the catalytic triad, is variable, with serovar Typhimurium SpvD having an arginine and serovar Enteritidis a glycine at this position. This variation affected hydrolytic activity of the enzyme on artificial substrates and can be explained by substrate accessibility to the active site. Interestingly, the SpvD^G161^ variant more potently inhibited NF-κB-mediated immune responses in cells *in vitro* and increased virulence of serovar Typhimurium in mice. In summary, our results explain the biochemical basis for the effect of virulence protein SpvD and demonstrate that a single amino acid polymorphism can affect the overall virulence of a bacterial pathogen in its host.

## Introduction

*Salmonella enterica* serovars Typhimurium and Enteritidis are broad host range intracellular bacterial pathogens causing gastrointestinal disease in humans and a typhoid-like systemic infection in certain mouse strains. Their virulence depends on the activity of two type III secretion systems (T3SSs),^5^ encoded by the pathogenicity islands *Salmonella* pathogenicity island 1 (SPI-1) and SPI-2 that enable translocation of effector proteins into host cells. The majority of effector proteins are encoded on the bacterial chromosome, but some are present on the pSLT virulence plasmid (1). An important virulence determinant of the pSLT plasmid is the *Salmonella* plasmid virulence locus (*spv*) (2–5), which comprises the *spvABCD* operon and its upstream regulator *spvR* (6). SpvB is an ADP ribosyl transferase that depolymerizes F-actin (7), and SpvC is a phosphothreonine lyase that inhibits mitogen-activated protein kinase signaling (8, 9). Mass spectrometry analysis of proteins secreted by *Salmonella* identified SpvD as an effector of both SPI-1 and SPI-2 T3SSs (10). Previously, we confirmed that SpvD contributes to virulence during systemic infection of mice (10, 11) and showed it inhibits the NF-κB signaling pathway and secretion of proinflammatory cytokines from infected macrophages (11). In this work we determined the structure of SpvD and showed that it is a hydrolase with a papain-like fold and a catalytic triad composed of Cys-73, His-162, and Asp-182. Although SpvD is highly conserved among different serovars of *S. enterica,* serovars Typhimurium and Enteritidis differ in containing an arginine or glycine, respectively, at residue 161 near the active site. We show that this polymorphism affects hydrolytic activity, degree of inhibition of the NF-κB pathway, and remarkably, bacterial virulence in mice.

## Results

### 

#### 

##### SpvD Adopts a Papain-like Fold

**FIGURE 1.**
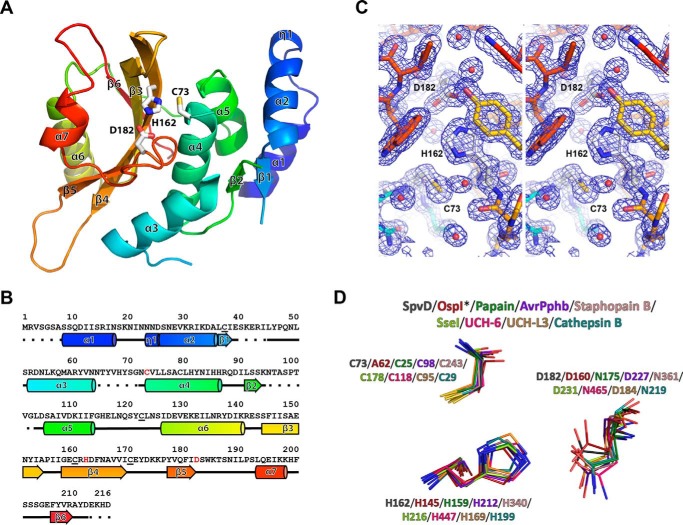
**SpvD adopted a papain-like fold with Cys-73, His-162, and Asp-182 catalytic triad.** *A*, crystal structure of *S. typhimurium* SpvD^4CS^ colored starting from N terminus (*blue*) and ending at C terminus (*red*). Secondary structure elements and catalytic triad (*sticks*) are labeled in *black*. Cys-73/His-162/Asp-182 catalytic triad is shown in *sticks*. The image was generated using PyMOL software. *B*, secondary structure elements mapped according to their respective amino acid sequence and colored as in *A*. In the sequence, the catalytic triad residues are colored in *red*. Surface cysteines mutated to serines are *underlined. C*, walleye stereo view of the 2*F_o_* − *F_c_* electron density map of the active site region of the SpvD structure. *D*, superimposition of catalytic triad components of SpvD, OspI^C62A^ (PDB code 3W31), papain (PDB code 1PPN), AvrPphB (PDB code 1UKF), staphopain B (PDB code 1Y4H), SseI (PDB code 4G29), UCH-6 (PDB code 1VJV), UCH-L3 (PDB code 1XD3), and cathepsin B (PDB code 3AI8).

**FIGURE 2.**
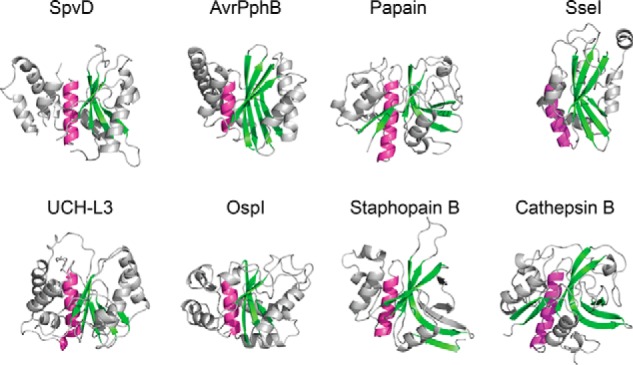
**SpvD structure shared papain-like fold features.** Schematic representation of SpvD^A154/R161^, OspI^C62A^ (PDB code 3W31), papain (PDB code 1PPN), AvrPphB (PDB code 1UKF), staphopain B (PDB code 1Y4H), SseI (PDB code 4G29), UCH-L3 (PDB code 1XD3), and cathepsin B (PDB code 3AI8) is shown. The central α-helix containing the catalytic cysteine is colored in *purple*, and the main anti-parallel β-sheet is colored in *green*. Structures were oriented according to their superimposed catalytic triads.

To gain insight into the function of SpvD, we attempted to determine its crystal structure. After unsuccessful crystallization of wild-type SpvD, we obtained crystals of SpvD in which its predicted surface cysteines were mutated (SpvD^C37S/C122S/C160S/C170S^, hereafter referred to as SpvD^4CS^), and these diffracted to 1.5 Å resolution (Table 1). Subsequent structure determination revealed that SpvD adopts an α+β fold with a total of 7 α-helices, 6 β-strands, and a short stretch of 3_10_ helix (Fig. 1, *A* and *B*). Its central core is composed of a β-sheet formed by four antiparallel β-strands (β3–6) surrounded by five α-helices (α3–7). Additionally, there is a smaller β-sheet (β1–2) surrounded by α-helices 1–5 (Fig. 1, *A* and *B*). Close inspection of a shallow groove on the protein surface between helices α4/α5, strand β4, and the β5-α7 loop revealed a potential hydrolase active site including a catalytic triad of cysteine (Cys-73), histidine (His-162), and aspartic acid (Asp-182) (Fig. 1, *A* and *C*). A structure similarity search performed with the DALI software (12) revealed similarities to four other secreted effector proteins: OspI (a T3SS deamidase of the Gram-negative bacterial pathogen *Shigella flexneri*) (13, 14), SseI (a *S. typhimurium* SPI-2 T3SS effector protein of unknown enzymatic activity) (15), AvrPphB (a T3SS cysteine protease of a bacterial plant pathogen *Pseudomonas syringae*) (16), and SspB (a cysteine protease secreted by *Staphylococcus aureus*) (17) as well as other papain and papain-like cysteine proteases. These all have a characteristic central antiparallel β-sheet and catalytic cysteine-containing α-helix (Fig. 2). Superimposition of these structures revealed that the orientation of the catalytic triads in papain, OspI, AvrPphB, staphopain B, SseI, UCH-L3, UCH-6, and cathepsin B proteins are almost identical to that of SpvD (Fig. 1*D*) (14–20).

**TABLE 1:** **Data collection and refinement statistics** r.m.s.d., root mean square deviation; SIRAS, single isomorphous replacement with anomalous scattering (data set collected from crystals soaked in KAuCl_4_).

	SpvD^4CS/A154/R161^	SpvD^4CS/A154/G161/C73A^	SpvD (Au SIRAS)
**Data collection**			
Wavelength (Å)	0.97625	0.97949	0.92000
Space group	P21	P21	P21
Cell dimensions			
*a*, *b*, *c* (Å)	43.7, 51.81, 47.27	44, 51.97, 47.22	43.84, 52.11, 47.38
α, β, γ (°)	90, 107.41, 90	90, 107.7, 90	90, 107.84, 90
Resolution range (Å)	51.81–1.48 (1.56–1.48)*^a^*	51.97–1.60 (1.69–1.60)	41.73–2.25 (2.31–2.25)
Reflections, total	195,684 (21,204)	156,479 (23,249)	
Reflections, unique	33,404 (4614)	26,737 (3,893)	
*I*/σ*I*	12.1 (2.1)	10.4 (1.9)	18.1 (3.8)
*R*_merge_	0.076 (0.869)	0.104 (1.018)	0.108 (0.531)
Completeness (%)	99.1 (94.1)	99.4 (99.2)	100 (60.8)*^b^*
Multiplicity	5.9 (4.6)	5.9 (6.0)	6.4 (4.2)*^b^*

**Refinement**			
*R*_factor_/*R*_free_ (%)	16.96/20.15	16.32/19.73	
No. of atoms			
Protein	1,556	1,713	
Ligand/ion	1	18	
Water	182	205	
*B*_factors_			
Protein	22.07	19.96	
Ligand/ion	17.18	28.19	
Water	30.76	30.61	
r.m.s.d. bond length (Å)	0.0151	0.0164	
r.m.s.d. bond angle (°)	1.573	1.410	
Ramachandran favored/outliers (%)	98.9/0	98.1/0.5	

*^a^* Values in parentheses correspond to the highest resolution shell.

*^b^* Completeness and multiplicity values given for anomalous data.

##### A Naturally Occurring SpvD Variant with Enhanced Enzymatic Activity

**FIGURE 3.**
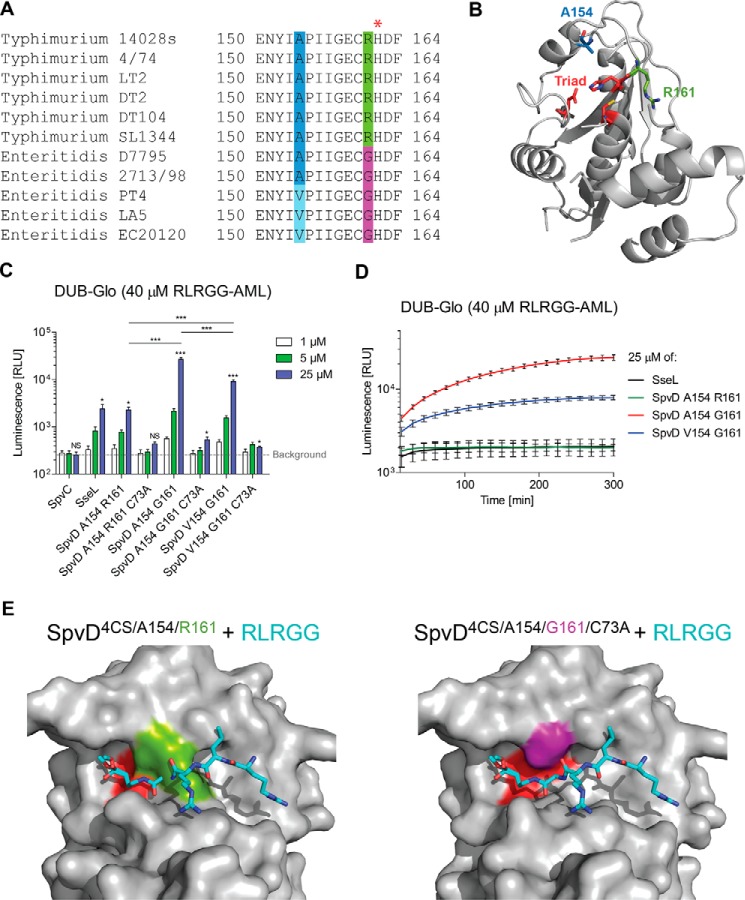
**SpvD had a deconjugative activity that varied between serovars.** *A*, zoom on SpvD amino acid alignment highlighting the two main (154 and 161) polymorphisms occurring in Enteritidis and Typhimurium serovars. The *red asterisk* indicates the catalytic histidine His-162. *B*, both Ala-154 (*blue stick*) and Arg-161 (*green stick*) positions are situated in the vicinity of the CHD catalytic triad (*red sticks*). *C*, DUB-Glo^TM^ assay with three (1, 5, and 25 μm) concentrations of tested proteins. Results are expressed as mean ± S.E. of three independent experiments. *Asterisks above the blue bars* represent significant differences when compared with the buffer control (background) as measured by the Student's *t* test. Comparison between SpvD variants at 25 μm concentration was made using one-way ANOVA statistical analysis with Bonferroni's multiple comparison test. *, *p* < 0.05; ***, *p* < 0.005; *NS*, not significant. *RLU*, relative light units. *D*, DUB-Glo^TM^ signal over time for the highest (25 μm) concentration tested for SseL, SpvD^A154/R161^, SpvD^A154/G161^, and SpvD^V154/G161^. Signal intensities were measured every 15 min for which ± S.E. of three independent experiments are shown. *E*, modeling of the RLRGG peptide onto the surface of SpvD^4CS/A154/R161^ (*left*) or SpvD^4CS/A154/G161/C73A^ (*right*) using the C-terminal ubiquitin tail (RLRGG; cyan sticks) from the UCH-L3 ubiquitin hydrolase complexed with ubiquitin (PDB code 1XD3). The catalytic triad region of SpvD is colored in *red*, Arg-161 is in *green*, and Gly-161 is in *purple*.

**FIGURE 4.**
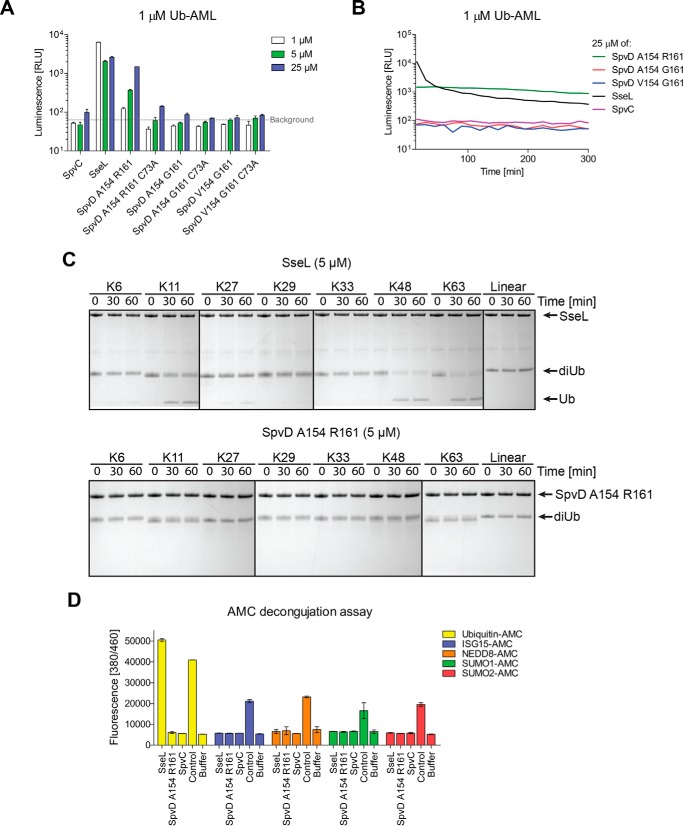
**SpvD^R161^ was active on a ubiquitin-AML substrate.** *A*, a modification of the DUB-Glo^TM^ assay with RLRGG substrate replaced by a full-length ubiquitin conjugated to aminoluciferin (*Ub-AML*). A representative assay result is shown. *B*, Ub-AML deconjugation assay over time measured for the highest concentration (25 μm) of the tested proteins. Signals were taken every 15 min. *C*, *in vitro* deubiquitination assay done with 5 μm of either SseL (*top*) or SpvD^A154/R161^ (*bottom*). Diubiquitin linkages were incubated with tested proteins for 0, 30, and 60 min followed by SDS-PAGE separation and Coomassie staining. Detectable cleavage product in the form of a single ubiquitin band is indicated with an *arrow. D*, AMC deconjugation assay using ubiquitin-AMC (*yellow*), ISG15-AMC (*blue*), NEDD8-AMC (*orange*), SUMO1-AMC (*green*), and SUMO2-AMC (*red*). Results are shown as mean fluorescence values with S.D. of three technical repeats obtained 1 h after start of the experiment. Isopeptidase T was used as a positive control for ubiquitin-AMC and ISG15-AMC. SENP2 was used as a control protease for SUMO1-AMC and SUMO2-AMC substrates, whereas NEDP1 was used in case of NEDD8-AMC.

The amino acid sequence of SpvD varies among serovars of *S. enterica* (21). We carried out a protein BLAST search using the amino acid sequence of *S. typhimurium* 14028 SpvD. This confirmed previously described polymorphisms and found sequences homologous to SpvD in other bacterial species such as *Pseudomonas psychrophila* and *Providencia burhodogranariea* (supplemental Fig. S1). Within the broad host range serovars of *S. enterica*, Enteritidis and Typhimurium, there are only two positions that consistently vary in amino acid composition (154 and 161) (Fig. 3*A* and supplemental Fig. S1). Within Typhimurium serovars, an alanine is encoded at position 154, and an arginine is encoded at position 161. In contrast, all Enteritidis serovars sequenced to date have either alanine or valine at position 154 and glycine at position 161 (Fig. 3*A*). Both residues are in close proximity to the catalytic triad, and their side chains contribute to the structure of the active site groove (Fig. 3*B*, *blue* and *green sticks*). Positions 154 and 161 of SpvD homologues in species other than *Salmonella* are mainly occupied by small hydrophobic residues (leucine and isoleucine at 154 and glycine and alanine at 161; supplemental Fig. S1). Within *Salmonella*, the Gly-161 polymorphism appears to be unique to Enteritidis, Abortus-equi, and Bovismorbificans serovars as all others (Typhimurium, Dublin, Paratyphi C, Gallinarum, Pullorum, and Choleraesuis) contain arginine at this position (supplemental Fig. S1). We used a variety of assays to determine the potential enzymatic activity of SpvD. Structural similarity to ubiquitin hydrolases suggested that SpvD could be a deubiquitinase. In a screen that utilizes a C-terminal peptide of ubiquitin (RLRGG) fused to aminoluciferin for detection of ubiquitin and ubiquitin-like cysteine proteases (DUB-Glo^TM^ protease assay; Promega), we found that SpvD, like the known *Salmonella* deubiquitinase (DUB) SseL (22, 23), produced a stable and specific luminescence signal indicative of substrate hydrolysis. Consistent with the crystal structure, SpvD hydrolytic activity was dependent on cysteine 73 of its putative catalytic triad (Fig. 3*C*). Interestingly, conversion of the SpvD sequence to that of the Enteritidis strains increased the activity observed in the DUB-Glo^TM^ protease assay (Fig. 3, *C* and *D*). At the highest concentration tested, the most active variant, SpvD^A154/G161^, produced over 10 times more luminescence signal than SpvD^A154/R161^, 5 h after the start of the assay (Fig. 3, *C* and *D*). In this assay, protease and luciferase activities normally reach a steady state so that a maximal signal is reached after 30 min and maintained for several hours. In contrast to SseL and SpvD^A154/R161^, the luminescence signal produced by the SpvD^A154/G161^ and SpvD^V154/G161^variants did not stabilize after 30 min but continued to increase up to 5 h (Fig. 3*D*). SpvD^V154/G161^ had an intermediate activity in the DUB-Glo^TM^ assay, in between that of SpvD^A154/R161^ and SpvD^A154/G161^ (Fig. 3*C* and *D*). Due to the more pronounced effect of the variation at position 161, we focused further studies on this polymorphism, maintaining alanine at position 154. To explain why the Gly-161 polymorphism leads to greater activity, we crystallized an inactive (C73A) form of the variant in the presence of RLRGG-AML substrate. Although density for the RLRGG-AML substrate was not observed, we were able to build additional loops (two of them near the active site) that were disordered in the previously obtained SpvD^4CS/R161^ structure (supplemental Fig. S2). To map a potential ligand binding site on SpvD, we superimposed the catalytic triad with that of the UCH-L3 ubiquitin hydrolase crystallized in complex with ubiquitin (Fig. 3*E*). The position of the superimposed five C-terminal amino acids of ubiquitin (RLRGG) is unobstructed in the SpvD^4CS/G161/C73A^ variant (Fig. 3*E*, *right*), whereas in SpvD^4CS/R161^, binding would be impeded by the side chain of Arg-161, which restricts access to the active site (Fig. 3*E*, *left*). Therefore, the Arg-161 side chain could have an inhibitory steric or regulatory role in the activity of SpvD. These results demonstrate that SpvD is a hydrolytic enzyme with a catalytic triad that is characteristic of papain-like proteases. As the DUB-Glo^TM^ protease assay detects ubiquitin and ubiquitin-like proteases, we then assayed SpvD variants using full-length ubiquitin-AML as a substrate. As expected, the positive control SseL was active at all concentrations tested (1 μm, 5 μm, and 25 μm; Fig. 4*A*). Surprisingly, the only SpvD variant that produced a specific, Cys-73-dependent signal was SpvD^A154/R161^ (Fig. 4, *A* and *B*). The luminescent signal produced by SseL decreased over time from an initial level of 100-fold above background, indicating substrate depletion (Fig. 4*B*). In contrast SpvD^A154/R161^produced a level of luminescence that was ∼10-fold above background and remained relatively constant over the course of the assay. Interestingly, in this assay both SpvD^V154/G161^ and SpvD^A154/G161^ displayed virtually no activity (Fig. 4, *A* and *B*). To test if SpvD^A154/R161^ has linkage-specific deubiquitinating activity, diubiquitin molecules linked by seven different lysine residues or methionine M1 were tested for cleavage. SseL cleaved isopeptide molecules linked by Lys-11, Lys-48, and Lys-63 (Fig. 4*C*, *top*), but SpvD^A154/R161^ had no detectable activity on any of the substrates (Fig. 4*C*, *bottom*). To test if SpvD^A154/R161^ has activity against other ubiquitin-like proteins, we used substrates comprising full-length ubiquitin or four ubiquitin-like proteins (SUMO1, SUMO2, NEDD8, and ISG15) C-terminally linked to 7-amino-4-methylcoumarin (AMC). SpvD^A154/R161^ had no detectable activity on any of these substrates, whereas SseL produced a specific fluorescent signal with ubiquitin-AMC as a substrate (Fig. 4*D*).

##### Inhibition of the NF-κB Pathway by SpvD Is Dependent on Cys-73 and Is Affected by Variation at Residue 161

**FIGURE 5.**
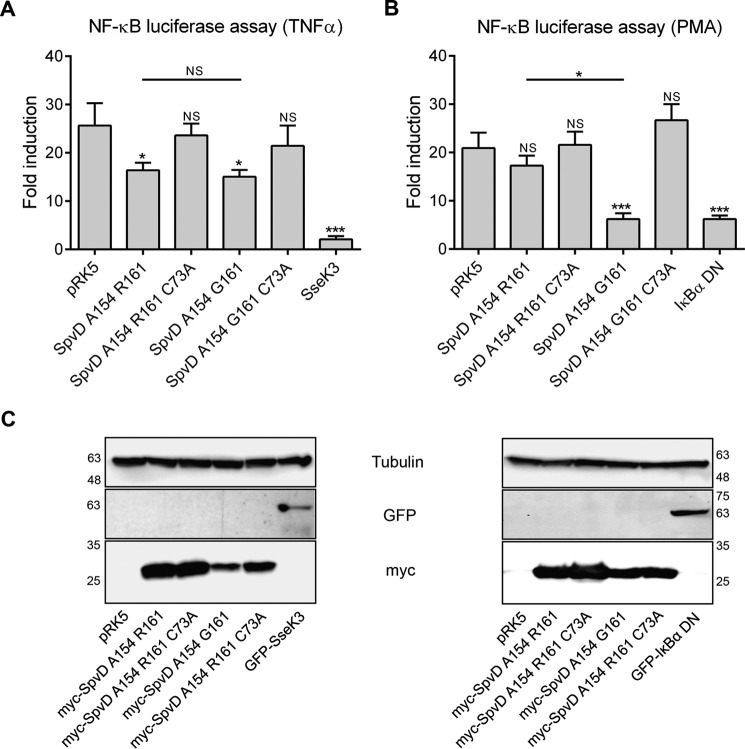
**SpvD variants had differential NF-κB inhibitory activities in PMA- but not in TNFα-stimulated cells.** NF-κB luciferase assay from transfected HEK293 cell line after 16 h stimulation with TNFα (*A*) or PMA (*B*). Results are shown as -fold activation in relation to unstimulated cells. Values are expressed as the mean ± S.E. of at least four independent experiments. Statistical significances were calculated using one-way ANOVA followed by Bonferroni's multiple comparison test against pRK5- or SpvD^A154/R161^-transfected cells (*, *p* < 0.05; ***, *p* < 0.005; *NS*, not significant). *C*, protein expression levels measured by SDS-PAGE and immunoblotting with anti-tubulin (*top*), anti-GFP (mid), and anti-myc (*bottom*) antibodies. *Numbers on the sides* correspond to molecular weight protein ladder in kDa.

We showed previously that SpvD inhibits the NF-κB signaling pathway (11). To investigate the influence of the amino acid side chain at position 161 and the requirement for the putative catalytic cysteine (Cys-73) on the anti-inflammatory activity of SpvD, HEK 293 cells were co-transfected with a reporter plasmid encoding luciferase under the control of an NF-κB promoter and a plasmid encoding either myc alone (pRK5 vector), myc-SpvD variants with alanine at position 154, or GFP-tagged positive control inhibitory proteins: *Salmonella* SseK3 (24) and NF-κB inhibitor α (IκBα) S32A/S36A mutant protein (25). Luciferase activity was measured 16 h after stimulation with TNFα or phorbol 12-myristate 13-acetate (PMA) and normalized to that of non-stimulated cells. TNFα and PMA caused ∼25- and 20-fold increases in NF-κB activation, respectively (Fig. 5, *A* and *B*). Expression of tagged proteins was confirmed by Western blotting (Fig. 5*C*). SseK3 is an effector that is known to inhibit the NF-κB pathway upon TNFα stimulation (14). In this assay it inhibited the reporter activity by 92% (Fig. 5*A*). After TNFα stimulation, SpvD^A154/R161^ and SpvD^A154/G161^ reduced NF-κB fold activation by 36 and 41%, respectively, and these effects were dependent on Cys-73 (Fig. 5*A*). There was no significant difference in the level of NF-κB inhibition between the Arg-161 and Gly-161 variants (Fig. 5*A*). However, after stimulation with PMA, both the positive control protein IκBα^S32A/S36A^ and SpvD^A154/G161^ inhibited the NF-κB pathway to a similar level (Fig. 5*B*), whereas SpvD^A154/R161^ had a much smaller effect. Therefore, the increased hydrolase activity of SpvD^A154/G161^ over SpvD^A154/R161^ in the DUB-Glo^TM^ protease assay (Fig. 3*C*) correlates with an enhanced ability of SpvD^G161^ to inhibit the NF-κB inflammatory pathway in cells stimulated with PMA.

##### Higher in Vitro Deconjugative and Anti-inflammatory Activities of Naturally Occurring SpvD Variant Correlate with Salmonella Virulence in Vivo

**FIGURE 6.**
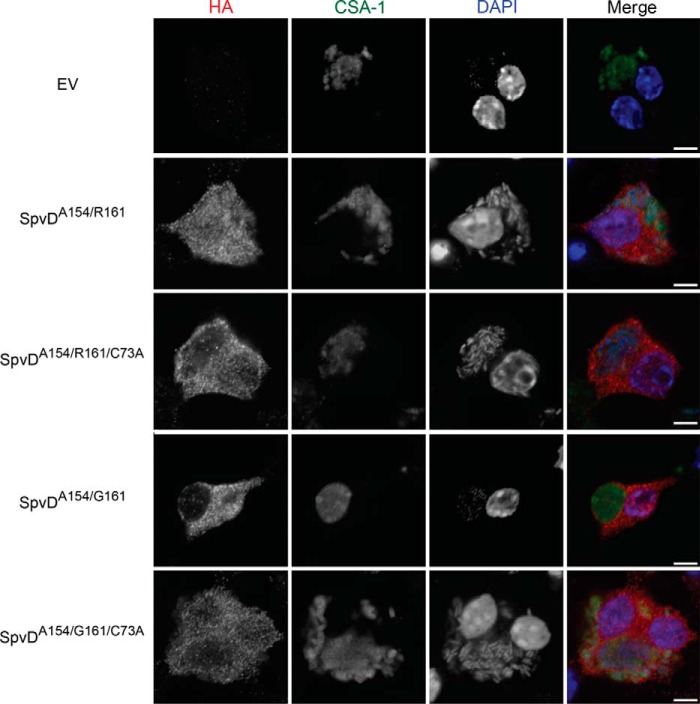
**Catalytically dead variants of SpvD were translocated from bacteria in iBMDMs.** Cells were infected for 18 h with *S. typhimurium spvD* strain carrying either an empty pWSK29 plasmid (*EV*) or a pWSK29 plasmid encoding C-terminally 2×HA-tagged variants of SpvD under SPI-2 promoter. In the last 2 h of infection, cells were treated with 10 μm MG-132 proteasomal inhibitor. DNA, *Salmonella*, and HA-tagged SpvD were stained using DAPI dye, CSA-1, and HA11 antibodies, respectively. The *white scale bar* represents 5 μm.

**FIGURE 7.**
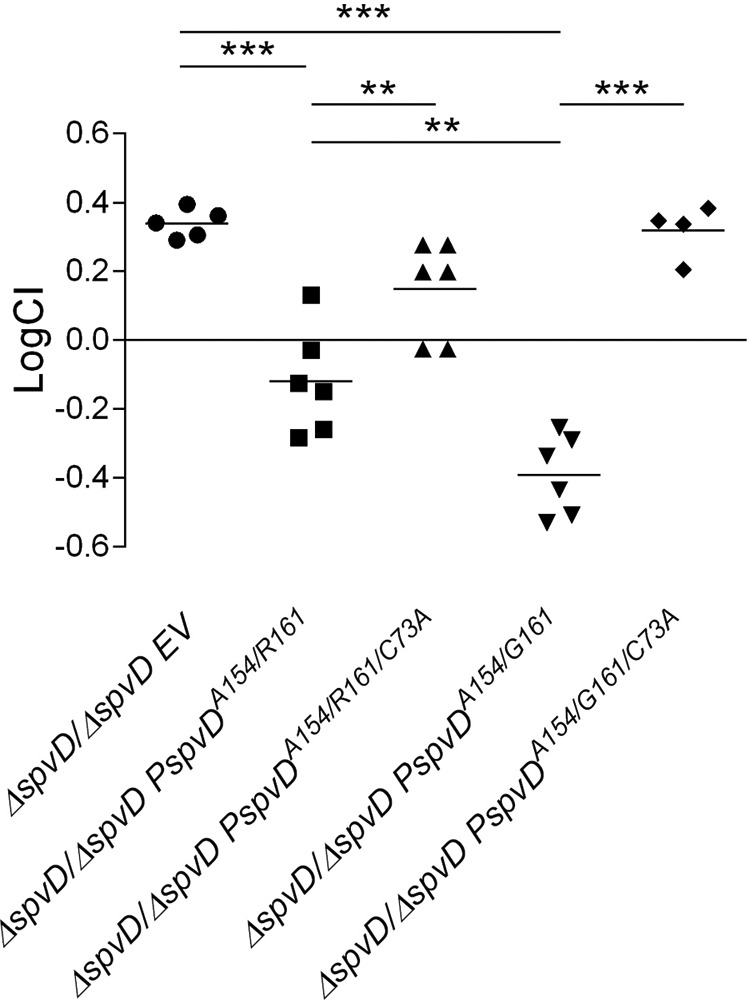
**SpvD^A154/G161^ conferred increased virulence over SpvD^A154/R161^.** C57BL/6 mice were inoculated by intraperitoneal injection with equal numbers (5 × 10^2^ cfu of each of the two strains) of the indicated bacteria. Bacteria were recovered from infected spleens 3 days post-inoculation. CI values were calculated as described under “Experimental Procedures.” The scatter plot displays values obtained for individual mice and the mean are indicated (*line*). Statistical significances were calculated using one-way ANOVA followed by Bonferroni's multiple comparison test. **, *p* < 0.01; ***, *p* < 0.005.

*Salmonella* strains lacking SpvD were shown to be attenuated for growth in mice when compared with the wild-type strain (10, 11). Having detected different *in vitro* deconjugation and anti-inflammatory activities of SpvD variants, we tested if the side chain at position 161 within the SpvD active site influences *Salmonella* growth *in vivo*. First, we confirmed bacterial translocation of C-terminal double hemagglutinin (HA)-tagged SpvD^A154/G161/C73A^ and SpvD^A154/R161/C73A^ into iBMDMs, showing that catalytic triad mutations did not affect effector delivery (Fig. 6). Mice were then inoculated by intraperitoneal injection with a mixture of equivalent cfu of Δ*spvD* mutant strain and chloramphenicol-resistant Δ*spvD* mutant strains harboring various pACYC184-based complementation plasmids (Fig. 7). After 72 h of infection, bacteria were quantified after plating spleen homogenates on LB agar medium and LB agar medium containing chloramphenicol to distinguish the strains. The competitive index (CI) of Δ*spvD* mutant and Δ*spvD* mutant harboring an empty pACYC184 plasmid (EV) was 0.34 ± 0.04, showing that the presence of pACYC184 incurred a fitness cost (Fig. 7). When mixed with Δ*spvD* strains carrying pACYC184 encoding either SpvD^A154/R161^ or SpvD^A154/G161^, the CI of the Δ*spvD* mutant strain dropped from 0.34 to −0.12 and −0.39, respectively (Fig. 7). Interestingly, the Δ*spvD* mutant was more efficiently outcompeted by the SpvD^A154/G161^ strain compared with the SpvD^A154/R161^ strain, demonstrating that the more active SpvD^A154/G161^ variant is more potent in complementing the Δ*spvD* mutation than SpvD^A154/R161^ (Fig. 7). When mixed with Δ*spvD* strains carrying plasmids encoding the C73A mutant versions of SpvD, the Δ*spvD* strain had CIs of 0.15 ± 0.14 and 0.32 ± 0.08 for SpvD^A154/R161/C73A^ and SpvD^A154/G161/C73A^, respectively) similar to the CI of the Δ*spvD* strain mixed with Δ*spvD* strain harboring empty pACYC184 plasmid (Fig. 7). Therefore, the ability of both SpvD^A154/R161^ and SpvD^A154/G161^ to complement the virulence defect was dependent on a functional catalytic triad.

## Discussion

In this work we have determined the structure of SpvD, a *S. typhimurium* SPI-2 T3SS effector protein that inhibits the NF-κB signaling pathway (11). Structurally, SpvD can be classified as belonging to the CA clan of cysteine proteases that contains papain and related proteases (MEROPS peptidase database). A hallmark of this clan is a CHD (or CHN) catalytic triad, which in the case of SpvD is composed of Cys-73, His-162, and Asp-182. Using a DUB-Glo^TM^ assay, we showed that SpvD is an enzyme with deconjugative activity. Taken together, the results of our experiments strongly suggest that SpvD acts as a protease. Due to the chemical nature of the DUB-Glo^TM^ substrate (aminoluciferin fused to the C terminus of an RLRGG peptide), the DUB-Glo^TM^ assay would potentially not only detect deubiquitinases or ubiquitin-like proteases but any protease that recognizes and cleaves after a sequence similar to GG. In all the assays tested (DUB-Glo^TM^, NF-κB inhibition, virulence in mouse) the activity of SpvD was abolished by a C73A mutation, demonstrating the necessity of an intact catalytic triad for its function. Previously, we reported that SpvD specifically interacts with exportin-2 to inhibit nuclear transport of p65 (11). Although the results presented here provide additional evidence for a function of SpvD in inhibiting the NF-κB pathway, we have not been able to detect cleavage of endogenous exportin-2 in HEK293 cells transiently expressing SpvD^A154/R161^ or SpvD^A154/G161^ (data not shown). This suggests that there might be another as yet unidentified substrate that is modified by SpvD. Analysis of naturally occurring SpvD polymorphisms revealed that position 161 is critical for the deconjugative activity of SpvD in the DUB-Glo^TM^ assay. Modeling of the RLRGG DUB-Glo^TM^ substrate onto the Arg-161 or Gly-161 structures provides a rationale for this observation, clearly showing that the side chain at position 161 forms part of the active site groove and that an arginine side chain here restricts access to the catalytic triad. Despite being less active in the DUB-Glo^TM^ assay, SpvD^A154/R161^ was the most active SpvD variant in the Ub-AML deconjugation assay. This suggested that SpvD^A154/R161^ might be a DUB or ubiquitin-like protease. However, we were unable to obtain evidence for this in subsequent AMC deconjugation and *in vitro* deubiquitination assays. When compared with the Typhimurium SpvD^A154/R161^ variant, SpvD with a single polymorphism (Gly-161) was more potent in (i) the DUB-Glo^TM^ protease assay, (ii) inhibition of the NF-κB luciferase assay in PMA-stimulated cells, and (iii) virulence in mice. The observation that SpvD^A154/G161^ is more potent at inhibiting NF-κB signaling than SpvD^A154/R161^ in PMA- but not TNFα-stimulated HEK293 cells might help guide the identification of its substrate. PMA mimics diacylglycerol in stimulating NF-κB through activation of protein kinase C (26). Therefore, our results suggest that the more active SpvD variant targets a diacylglycerol-stimulated branch of NF-κB pathway. There are very few descriptions of single amino acid polymorphisms influencing the potency of bacterial effectors. A naturally occurring polymorphism within *S. typhimurium* SseI at position 103 was shown to affect binding to the cell migration adaptor protein TRIP6 in a yeast two-hybrid assay and bacterial dissemination in mice (27). However, the position of the SseI polymorphism in relation to the catalytic site is unknown as only partial SseI (amino acids 145–313) structure is currently available (15). The SpvD polymorphism that we have investigated here is in very close vicinity of the catalytic triad. Depending on the substrate used *in vitro*, the Arg-161 or Gly-161 variant displayed greater potency. However, the Gly-161 variant conferred stronger inhibition of a PMA-stimulated NF-κB reporter and greater virulence of *Salmonella* in mice. Regardless of the identity of the physiological substrate for hydrolytic activity of SpvD, it is remarkable that a single amino acid substitution that alters hydrolytic potency can influence the complex process of virulence in a host. The fact that the SpvD^R161^ variant is present in all *Salmonella* serovars except Enteritidis, Bovismorbificans, and Abortus-equi is intriguing. It is possible that SpvD^R161^ has gained a different substrate specificity that is not related to NF-κB signaling. Collectively, our results indicate SpvD is a papain-like hydrolase that functions through a cysteine-mediated hydrolysis reaction to inhibit gene expression from NF-κB promoters. Furthermore, we demonstrate that a natural SpvD polymorphism significantly affects virulence and NF-κB inhibition in PMA-stimulated cells, leading us to hypothesize that the substrate hydrolyzed by SpvD^G161^ might be part of a signaling pathway involving protein kinase C.

## Experimental Procedures

### 

#### 

##### Bacterial Strains and Plasmids

Bacterial strains and plasmids used in this study are listed in supplemental Table S1. The *Salmonella* strains were grown in Luria Bertani (LB) medium at 37 °C with shaking and supplemented with ampicillin (100 μg/ml) or chloramphenicol (34 μg/ml) as appropriate. Complementation plasmids (pACYC184 or pWSK29) containing non-tagged or double HA-tagged *spvD* were obtained using restriction enzyme cloning. Site-directed mutagenesis of SpvD was performed by inverse PCR using pRK5-myc-SpvD^A154/R161^ vector as DNA template.

##### Protein Production

For crystallography, SpvD^C73S/C122S/C160S/C170S^ and SpvD^C73S/C122S/C160S/C170S/C73A/G161^ were expressed with a precision protease-cleavable N-terminal His_6_ tag from pHiSH, a pET15b-derived vector (28) in PC2 cells (29). Cultures were grown in Terrific Broth or LB to an *A*_600_ of 0.8 at 37 °C and induced with 0.5 mm isopropyl 1-thio-β-d-galactopyranoside for 16 h at 18 °C. Cells were lysed by sonication in 50 mm Tris/Cl, pH 7.5, 500 mm NaCl, 20 mm imidazole, 0.5 mm phenylmethylsulfonyl fluoride. After clarification by centrifugation, the lysate was mixed with nickel-nitrilotriacetic acid resin for 1 h at 4 °C. The resin was extensively washed with 50 mm Tris/Cl, pH 7.5, 500 mm NaCl, and 20 mm imidazole, and SpvD was eluted with 50 mm Tris/Cl, pH 7.5, 500 mm NaCl, and 500 mm imidazole. The His tag was removed by the addition of 10 mm dithiothreitol and HRV 3C protease at a ratio of 1 mg protease to 30 mg SpvD and overnight incubation at 4 °C. The SpvD solution was loaded onto a Superdex75 gel filtration column (GE Healthcare) pre-equilibrated in 50 mm Tris/Cl, pH7.5, 150 mm NaCl, and 10 mm β-mercaptoethanol. For biochemical analyses, GST-SseL, SpvC-His_6_, and SpvD^A154/R161^-His_6_ proteins were purified from previously generated pGEX and pET22b vectors (9, 11, 22). Variants of SpvD were made using site-directed mutagenesis, and their protein-coding DNA sequences were ligated into pET22b using identical restriction sites. Protein was expressed in *Escherichia coli* BL21(DE3) cells that were grown to an *A*_600_ of 0.8–1.0 at 37 °C and induced with 1 mm isopropyl 1-thio-β-d-galactopyranoside for 16–20 h at 20 °C. Depending on the protein fusion tag, proteins were purified to apparent homogeneity using either nickel- or glutathione-based affinity chromatography as described previously (9, 22).

##### DUB-Glo^TM^ and Ub-AML Assays

Purified SpvC, SseL, and SpvD proteins were each used in the DUB-Glo^TM^ assay at 1, 5, and 25 μm concentrations. The DUB-Glo^TM^ assay was performed using the manufacturer's instructions (Promega). Briefly, 50 μl of 2, 10, and 50 μm of tested proteins diluted in reaction buffer (50 mm HEPES, pH 7.4, 50 mm NaCl, 0.5 mm EDTA, 10 mm DTT) was mixed with 50 μl of RLRGG-AML substrate containing luciferase reporter enzyme. Luciferase activity was measured at 22 °C every 15 min up to 5 h with an Infinite M200Pro plate reader (Tecan). In a modification of the assay, 40 μm RLRGG-AML was replaced with 1 μm Ub-AML, whereas the remaining buffer composition and luciferase enzyme remained unchanged.

##### AMC Deconjugation Assay

SseL, SpvC, SpvD^A154/R161^, and relevant control proteins were incubated with 1 μm concentrations of ubiquitin-, SUMO1-, SUMO2-, ISG15-, and NEDD8-AMC (Boston Biochem) in 50 mm HEPES, pH 8.0, and 1 mm DTT. Liberation of AMC at 22 °C was measured after 1 h of incubation using Infinite M200Pro plate reader (Tecan; excitation/emission wavelengths 380/460 nm).

##### In Vitro Deubiquitination Assay

A panel of M1 linear Lys-6-, Lys-11-, Lys-27-, Lys-29-, Lys-33-, Lys-48-, and Lys-63-linked diubiquitin chains was acquired from UbiQ. The reaction mixture contained 50 mm HEPES, pH 7.5, 100 mm NaCl, 1 mm EDTA, 5 mm DTT, and 5 μm diubiquitin chains. Diubiquitin hydrolysis reactions were performed at 37 °C by adding 5 μm concentrations of either SseL (positive control) or SpvD. The assay was started by mixing the tested protein and diubiquitin solutions, and 10-μl aliquots were taken at the indicated time points. The reaction was resolved on 4–12% SDS-PAGE gradient gels run in MES buffer (Invitrogen) and stained with Coomassie Brilliant Blue R-250 (Bio-Rad).

##### Antibodies

Immunofluorescence analysis was done using goat anti-*Salmonella* (CSA-1, Kirkegaard and Perry Laboratories) and mouse anti-HA (HA11, Covance) antibodies. Immunoblotting of tagged proteins was performed using 9e10 (Roche Applied Science) and GFP (Life Technologies) antibodies. Tubulin was probed with E7 antibody (Developmental Studies Hybridoma Bank).

##### Cell Culture

HEK293 (human embryonic kidney) cells used in this study were obtained from the European Collection of Animal and Cell Cultures (Salisbury, UK) and maintained in Dulbecco's modified Eagle's medium (DMEM) (Life Technologies) supplemented with 10% fetal calf serum (FCS) (PAA Laboratories or Sigma) at 37 °C in 5% CO_2_. Bone marrow-derived macrophages (BMDM) were infected with the v-myc/v-raf expressing J2 retrovirus (30) and differentiated in 20% LCM (L929 cell (ATCC) conditioned medium). Cells were then maintained in DMEM (Sigma), 10% FCS, 20% LCM, and 1 mm sodium pyruvate at 37 °C, 5% CO_2_.

##### Crystallization and Structure Determination

SpvD^4CS^ and SpvD^4CS/C73A/G161^ proteins at 9 mg/ml in 50 mm Tris/Cl, pH 7.5, 50 mm NaCl, and 10 mm β-mercaptoethanol were crystallized by vapor diffusion in hanging drops against a reservoir of 100 mm Tris/Cl, pH 7.5, 200 mm NaCl, 22% PEG3350 at 4 °C. Two different crystal morphologies grew in the drops, and seeding was used to propagate the three-dimensional crystal form, which diffracted consistently to high resolution. SpvD^4CS^ crystals were transferred to a cryoprotectant solution of 100 mm Tris/Cl, pH 7.5, 200 mm NaCl, 22% PEG3350, and 20% glycerol and flash-frozen in liquid nitrogen, and diffraction data were collected to 1.48 Å on beamline I03 at Diamond Light Source (Oxfordshire, UK) (Table 1). Anomalous data were collected on I04-1 at Diamond Light Source from a crystal soaked for 1 h in 100 mm Tris/Cl, pH 7.5, 200 mm NaCl, 22% PEG3350, 20% glycerol, 1 mm KAuCl_4_. SpvD^4CS/C73A/G161^ crystals were grown as above and transferred stepwise with increasing concentrations of dimethyl sulfoxide (DMSO) and Z-RLRGG-aminoluciferin (Promega) to a final solution of 100 mm Tris/Cl, pH 7.5, 200 mm NaCl, 22% PEG3350, 25% DMSO, 1 mm Z-RLRGG-aminoluciferin before flash-freezing and collecting data at Diamond Light Source beamline I02. Native data were integrated in Mosflm (31) and merged in Scala (32) of the CCP4 suite (33), and anomalous data were processed with the xia2 pipeline using XDS and XSCALE (34–37). The structure was solved by single isomorphous replacement with anomalous scattering in Phenix using the AutoSol wizard (38, 39) with one site and an initial figure of merit of 0.35. The initial structure was built by Phenix AutoBuild wizard (40) and refined using Refmac and Phenix (38, 41) iterated with manual model building in Coot (42). Structures were refined to final resolutions of 1.48 Å (Arg-161) and 1.60 Å (Gly-161) with good geometry (Table 1). The coordinates and structure factors of the structures of SpvD^4CS/R161^ and SpvD^4CS/C73A/G161^ have been deposited in the protein data bank with PDB codes 5LQ6 and 5LQ7, respectively.

##### DNA Transfection

HEK293 cells were seeded in 24-well plates at a concentration of 2 × 10^4^ cells/well 24 h before transfection with Lipofectamine 2000 (Life Technologies) following the manufacturer's protocol. Cells were used 24 h after transfection.

##### Fixation, Permeabilization, Fluorescence Labeling, and Microscopy

All samples were fixed in 3% paraformaldehyde. Permeabilization was done in 0.2% Triton X-100 together with incubation with primary antibodies. All antibodies were diluted to the appropriate concentrations in PBS containing 10% horse serum and 0.2% Triton-X100. The coverslips were washed once in PBS, incubated with primary antibodies for 2 h, washed 3 times in PBS, and incubated with secondary antibodies and nucleic acid dye DAPI (Thermo Fisher) for 1 h. Coverslips were washed and mounted onto glass slides using Mowiol mounting medium. Cells were analyzed using a confocal laser-scanning microscope (LSM710; Zeiss GmbH).

##### Luciferase Reporter Assay

HEK293 cells were seeded at a density of 5 × 10^4^ cells per well in a 24-well plate 24 h before transfection. Cells were transfected for 24 h with 50 ng of luciferase reporter plasmid (NF-κB dependent luciferase reporter plasmid), 30 ng of pTK-Renilla luciferase, and 500 ng of expression vectors (SpvD, SseK3, IκBα^S32A/S36A^ or pRK5 vector alone). Cells were then incubated either with 10 ng/ml TNFα or 100 nm PMA for 16 h and harvested in 100 μl of passive lysis buffer (Promega). Luciferase activity was measured using the Dual Luciferase reporter assay system (Promega) and Infinite M200Pro plate reader (Tecan) and normalized according to Renilla luciferase intensity. The data presented are from at least four independent experiments.

##### Mice Infections

To prepare the inocula, bacteria were first grown overnight in LB broth and then subcultured at a dilution of 1:100 for a further 2 h. Cultures were diluted to a concentration of 5 × 10^3^ cfu/ml in physiological saline. For CI measurements, bacterial cultures were mixed for intraperitoneal inoculation (0.2 ml per mouse). Viable bacteria in inocula were quantified by dilution and plating onto LB agar plates with appropriate antibiotics to distinguish between strains. Female C57BL/6 mice (Charles River, 6–8 weeks) were sacrificed at 3 days post inoculation. The spleens were removed aseptically and homogenized in distilled water by mechanical disruption. Serial dilutions were plated on LB agar for cfu enumeration. Strains were distinguished by differential counting or replica plating on antibiotic-supplemented plates. For each mouse, the CI was calculated by dividing the output ratio (*i.e.* strain a *versus* strain b) divided by the input ratio. The log CI values were used to calculate means and for statistical analyses.

##### Ethics Statement

Animals were used in accordance with UK Home Office regulations. The Imperial College Animal Welfare and Ethical Review Body (AWERB) committee approved the project license for animal research (70/7768). The following people formed the panel: Applicant Scientist, CBS site manager/NACWO, NVS, peer scientist, and a lay person.

##### Statistical Analysis

All results are reported as the mean ± S.E. Statistical analyses were done using one-tailed paired Student's *t* test or ANOVA followed by Bonferroni's multiple comparison test. Differences denoted in the text as significant represent *p* values lower than 0.05.

## Author Contributions

G. J. G., S. A. H., and D. W. H. designed the project. N. R. and Y. Y. designed and purified SpvD4CS. Y. Z., M. P., and S. A. H. performed crystal structures determination. J. N. P. and D. K. provided the amino acid sequence and structural insights. G. J. G. performed SpvD activity assays, immunofluorescence, and mouse infections. G. J. G., S. A. H., and D. W. H. wrote the paper with contributions from the other authors.

## Supplementary Material

Supplemental Data

## Footnotes

5 The abbreviations used are:

T3SS: type III secretion system
SPI-1: *Salmonella* pathogenicity island 1
*spv*: plasmid virulence locus
DUB: deubiquitinase
4CS: SpvD^C37S/C122S/C160S/C170S^
AML: aminoluciferin
AMC: 7-amino-4-methylcoumarin
PMA: 12-myristate 13-acetate
CI: competitive index
(i)BMDM: (immortalized) bone marrow-derived macrophage
ANOVA: analysis of variance
Ub: ubiquitin.
