# Supplementary material for: The Salmonella Effector SpvD Is a Cysteine Hydrolase with a Serovar-specific Polymorphism Influencing Catalytic Activity, Suppression of Immune Responses, and Bacterial Virulence
Source: J Biol Chem. 2016 Oct 27;291(50):25853–63. doi: 10.1074/jbc.M116.752782 (PMC5207060; doi:10.1074/jbc.M116.752782)
Supplement: Supplemental Data [file supp_291_50_25853__index.html]

The Salmonella Effector SpvD is a Cysteine Hydrolase with a Serovar-Specific Polymorphism Influencing Catalytic Activity, Suppression of Immune Responses and Bacterial Virulence — The Salmonella Effector SpvD Is a Cysteine Hydrolase with a Serovar-specific Polymorphism Influencing Catalytic Activity, Suppression of Immune Responses, and Bacterial Virulence — SpvD Structure and Activity — Supplemental Data 

# The *Salmonella* Effector SpvD Is a Cysteine Hydrolase with a Serovar-specific Polymorphism Influencing Catalytic Activity, Suppression of Immune Responses, and Bacterial Virulence

## Supplemental Data

- Supplementary data (.pdf, 2.3 MB) - Supplementary data
