# Supplementary material for: The Salmonella Effector SpvD Is a Cysteine Hydrolase with a Serovar-specific Polymorphism Influencing Catalytic Activity, Suppression of Immune Responses, and Bacterial Virulence
Source: J Biol Chem. 2016 Oct 27;291(50):25853–63. doi: 10.1074/jbc.M116.752782 (PMC5207060; doi:10.1074/jbc.M116.752782)
Supplement: Supplemental Data [file 10.1074_M116.752782_jbc.M116.752782-1.pdf]

Table S1. Strains and plasmids used in this study

Name	Description	Reference
**Strains**		
S. Typhimurium		
ΔspvD	12023 *ΔspvD*	(11)
ΔspvD, *p*ACYC *(EV)*	*ΔspvD p*ACYC	This study
ΔspvD, *p*ACYCspvD^{R161}	*ΔspvD p*ACYCspvD^{R161}	(11)
ΔspvD, *p*ACYCspvD$^{R161/C73A}$	*ΔspvD p*ACYCspvD$^{R161/C73A}$	This study
ΔspvD, *p*ACYCspvD^{G161}	*ΔspvD p*ACYCspvD^{G161}	This study
ΔspvD, *p*ACYCspvD$^{G161/C73A}$	*ΔspvD p*ACYCspvD$^{G161/C73A}$	This study
ΔspvD, *pWSK29 (EV)*	*ΔspvD p*WSK29	This study
ΔspvD, *p*spvD^{R161}-2HA	*ΔspvD p*WSK29spvD^{R161}-2xHA	This study
ΔspvD, *p*spvD$^{R161/C73A}$-2xHA	*ΔspvD p*WSK29spvD$^{R161/C73A}$-2xHA	This study
ΔspvD, *p*spvD^{G161}-2xHA	*ΔspvD p*WSK29spvD^{G161}-2xHA	This study
ΔspvD, *p*spvD$^{G161/C73A}$-2xHA	*ΔspvD p*WSK29spvD$^{G161/C73A}$-2xHA	This study
E. coli		
DH5α	wt	NCTC
BL21(DE3)	wt	NCTC
PC2	wt	(29)
**Plasmids**		
pET22b	Protein expression vector used for expression and purification of SpvD and SpvC proteins	Merck Millipore
pHiSH	Protein expression vector with prescision protease cleavable His-tag for expressing proteins for crystallography	(28)
*p*ACYC184	Rep_{p15A} low copy number vector	(43)
*p*ACYCspvD and mutant derivatives	*p*ACYC184 with *spvD* cloned into *EcoRV* and *SalI* sites, Cm^{R}	This study
*p*GEX-4T-2sseL	GST fused to sseL	(22)
*p*WSK29SpvD-2xHA and mutant derivatives	*p*WSK29 with spvD containing C-terminal 2xHA tag, Amp^{R}	This study
*p*RK5-myc	Eukaryotic expression vector	Laboratory stock
*p*RK5-myc-SpvD and mutant derivatives	*p*RK5 with N-terminal myc-tagged SpvD cloned into *BamHI* and *EcoRI* sites, Amp^{R}	This study
ptCMV-IκBα$^{S32A/S36A}$	Positive NF-κB inhibition protein	Laboratory stock
ptCMV-GFP-SseK3	GFP-fused effector known to inhibit NF-kB in TNFα stimulated cells	Laboratory stock
M_3psinrevκB-*luc*	*luc* gene under control of NF-κB consensus promoter	Gift from Felix Randow
*p*RLTK	constitutively active Renilla luciferase	Gift from Felix Randow

```
Pseudomonas sp. CF149           1   ------------------------------------------------------MKVF------GPAAQDHV-----QALRFTQRQIEDNETHDLNRIKQSLHIATDP-SPLPEHLDIESLRQLASFTNFTYLHYSGNCCLL 76
Pseudomonas psychrophila        1   ------------------------------------------------------MKVF------GPAAQDHA-----QALRFTQRQIEDNETHDLNRIKQSLHIATDP-SPLPEHLDIESLRQLASFTNFTYLHYSGNCCLL 76
Providencia burhodogranariea    1   MPISTININSPRQNTRENNTGISDNIKLEFSSNIVAIKPVNKATKLRKVSNNFLERIFNNLNTNAINIQKEIHTVWLGNTKISNNEMTDSDFKDVIRIKDALCVKRKEKKLYPQTLRKNDLEKMAKYVNKPFIHYSGNCAIL 142
Bovismorbificans                1   ------------------------------------------------------MRV------SGSASSQD------IISRINSKNINNNDSNEVKRIKDALCIESKERILYPQNLSRDNLKQMARYVNNTYVHYSGNCVLL 76
Enteritidis LA5                 1   ------------------------------------------------------MRV------SGSASSQD------IISRINSKNINNNDSNEVKRIKDALCIESKERILYPQNLSRDNLKQMARYVNNTYVHYSGNCVLL 76
Enteritidis EC201200            1   ------------------------------------------------------MRV------SGSASSQD------IISRINSKNINNNDSNEVKRIKDALCIESKERILYPQNLSRDNLKQMARYVNNTYVHYSGNCVLL 76
Enteritidis PT4                 1   ------------------------------------------------------MRV------SGSASSQD------IISRINSKNINNNDSNEVKRIKDALCIESKERILYPQNLSRDNLKQMARYVNNTYVHYSGNCVLL 76
Enteritidis D7795               1   ------------------------------------------------------MRV------SGSASSQD------IISRINSKNINNNDSNEVKRIKDALCIESKERILYPQNLSRDNLKQMARYVNNTYVHYSGNCVLL 76
Enteritidis 2713/98             1   ------------------------------------------------------MRV------SGSASSQD------IISRINSKNINNNDSNEVKRIKDALCIESKERILYPQNLSRDNLKQMARYVNNTYVHYSGNCVLL 76
Abortus equi                    1   ------------------------------------------------------MRV------SGSASSQD------IISRINSKNINNNDSNEVKRIKDALCIESKERILYPQNLSRDNLKQMARYVNNTYVHYSGNCVLL 76
Paratyphi C                     1   ------------------------------------------------------MRV------SGSASSQD------IISRINSKNINNNDSNEVKRIKDALCIESKERILYPQNLSRDNLKQMARYVNNTYVHYSGNCVLL 76
Typhimurium DT104               1   ------------------------------------------------------MRV------SGSASSQD------IISRINSKNINNNDSNEVKRIKDALCIESKERILYPQNLSRDNLKQMARYVNNTYVHYSGNCVLL 76
Typhimurium 14028s              1   ------------------------------------------------------MRV------SGSASSQD------IISRINSKNINNNDSNEVKRIKDALCIESKERILYPQNLSRDNLKQMARYVNNTYVHYSGNCVLL 76
Typhimurium SL1344              1   ------------------------------------------------------MRV------SGSASSQD------IISRINSKNINNNDSNEVKRIKDALCIESKERILYPQNLSRDNLKQMARYVNNTYVHYSGNCVLL 76
Typhimurium 4/74                1   ------------------------------------------------------MRV------SGSASSQD------IISRINSKNINNNDSNEVKRIKDALCIESKERILYPQNLSRDNLKQMARYVNNTYVHYSGNCVLL 76
Typhimurium DT2                 1   ------------------------------------------------------MRV------SGSASSQD------IISRINSKNINNNDSNEVKRIKDALCIESKERILYPQNLSRDNLKQMARYVNNTYVHYSGNCVLL 76
Typhimurium LT2                 1   ------------------------------------------------------MRV------SGSASSQD------IISRINSKNINNNDSNEVKRIKDALCIESKERILYPQNLSRDNLKQMARYVNNTYVHYSGNCVLL 76
Dublin                          1   ------------------------------------------------------MRV------SGSASSQD------IISRINSKNINNNDSNEVKRIKDALCIESKERILYPQNLSRDNLKQMARYVNNTYVHYSGNCVLL 76
Choleraesuis RF-1               1   ------------------------------------------------------MRV------SGSASSQD------IISRINSKNINNNDSNDVKRIKDALCIESKERILYPQNLSRDNLKQMARYVNNTYVHYSGNCVLL 76
Choleraesuis OU7025             1   ------------------------------------------------------MRV------SGSASSQD------IISRINSKNINNNDSNEVKRIKDALCIESKERILYPQNLSRDNLKQMARYVNNTYIHYSGNCVLL 76
Pullorum                        1   ------------------------------------------------------MRV------SGSASSQN------IISRINSKNINNNDSNEVKRIKDALCIESKERILYPQNLSRDNLKQMARYVNNTYIHYSGNCVLL 76
Gallinarum 1910/93              1   ------------------------------------------------------MRV------SGSASSRN------IISRINSKNINNNDSNEVKRIKDALCIESKERILYPQNLSRDNLKQMARYVNNTYIHYSGNCVLL 76
Gallinarum 72/805               1   ------------------------------------------------------MRV------SGSASSQD------IISRINSKNINNNDSNEVKRIKDALCIESKERILYPKNLSLDNLKQMARYVNNTYIHYSGNCVLL 76
Typhimurium ST313               1   ------------------------------------------------------MRV------SGSASSQD------IISRINSKNINNNDSNEVKRIKDALCIESKERILYPQNLSRDNLKQMARYVNNTYVHYSGNCVLL 76

Pseudomonas sp. CF149           77  SACVHYNIENNASILRTENTSQPHIGLEGDVVDEVIFGKVLPSSYPLFSIDEVSAEVLRRYEVNGE-RSCIVSTYNYIIPLIGESGHDFNAVVVLDSQNKPAVQYLDAWKTSNTTPDSKALEARFPPSARFTLRSFGSV-- 214
Pseudomonas psychrophila        77  SACVHYNIENNASILRTENTSQPHIGLEGDVVDEVIFGKVLPSSYPLFSIDEVSAEVLRRYEVNGE-RSCIVSTYNYIIPLIGESGHDFNAVVVLDSQNKPAVQYLDAWKTSNTTPDSKALEARFPPSARFTLRSFGRV-- 214
Providencia burhodogranariea    143 SACLHYNIHYRQDIYSVKNTKSPTVGLESTLVDRVIFGQKLKLSYF-YSIDDVKNEVLRRYNLNKEDFSFIISAENYILPVIGECAHDFNAVIIQEKNKEPCVQFIDAWKTFNKLPTFGELKSYFPPSTLFFIRAYYENKQ 282
Bovismorbificans                77  SACLHYNIHHRQDILSSKNTASPTVGLDSAIVDKIIFGHELNQSYCLNSIDEVEKEILNRYDIKRE-SSFIISAENYIVPIIGECGHDFNAVVICEYDKKPYVQFIDSWKTSNILPSLQEIKKHFSSSGEFYVRAYDEKHD 216
Enteritidis LA5                 77  SACLHYNIHHRQDILSSKNTASPTVGLDSAIVDKIIFGHELNQSYCLNSIDEVEKEILNRYDIKRE-SSFIISAENYIVPIIGECGHDFNAVVICEYDKKPYVQFIDSWKTSNILPSLQEIKKHFSSSGEFYVRAYDEKHD 216
Enteritidis EC201200            77  SACLHYNIHHRQDILSSKNTASPTVGLDSAIVDKIIFGHELNQSYCLNSIDEVEKEILNRYDIKRE-SSFIISAENYIVPIIGECGHDFNAVVICEYDKKPYVQFIDSWKTSNILPSLQEIKKHFSSSGEFYVRAYDEKHD 216
Enteritidis PT4                 77  SACLHYNIHHRQDILSSKNTASPTVGLDSAIVDKIIFGHELNQSYCLNSIDEVEKEILNRYDIKRE-SSFIISAENYIVPIIGECGHDFNAVVICEYDKKPYVQFIDSWKTSNILPSLQEIKKHFSSSGEFYVRAYDEKHD 216
Enteritidis D7795               77  SACLHYNIHHRQDILSSKNTASPTVGLDSAIVDKIIFGHELNQSYCLNSIDEVEKEILNRYDIKRE-SSFIISAENYIAPIIGECGHDFNAVVICEYDKKPYVQFIDSWKTSNILPSLQEIKKHFSSSGEFYVRAYDEKHD 216
Enteritidis 2713/98             77  SACLHYNIHHRQDILSSKNTASPTVGLDSAIVDKIIFGHELNQSYCLNSIDEVEKEILNRYDIKRE-SSFIISAENYIAPIIGECGHDFNAVVICEYDKKPYVQFIDSWKTSNILPSLQEIKKHLSSSGEFYVRAYDEKHD 216
Abortus equi                    77  SACLHYNIHHRQDILSSKNTASPTVGLDSAIVDKIIFGHELNQSYCLNSIDEVEKEILNRYDIKRE-SSFIISADNYIAPIIGECGHDFNAVVICEYDKKPYVQFIDSWKTSNILPSLQEIKKHFSSSGEFYVRAYDEKHD 216
Paratyphi C                     77  SACLHYNIHHRQDILSSKNTASPTVGLDSAIVDKIIFGHELNQSYCLNSIDEVEKEILNRYDIKRE-SSFIISAENYIVPIIGECRHDFNAVVICEYDKKPYVQFIDSWKTSNILPSLQEIKKHFSSSGEFYVRAYDEKHD 216
Typhimurium DT104               77  SACLHYNIHHRQDILSSKNTASPTVGLDSAIVDKIIFGHELNQSYCLNSIDEVEKEILNRYDIKRE-SSFIISAENYIAPIIGECRHDFNAVVICEYDKKPYVQFIDSWKTSNILPSLQEIKKHFSSSGEFYVRAYDEKHD 216
Typhimurium 14028s              77  SACLHYNIHHRQDILSSKNTASPTVGLDSAIVDKIIFGHELNQSYCLNSIDEVEKEILNRYDIKRE-SSFIISAENYIAPIIGECRHDFNAVVICEYDKKPYVQFIDSWKTSNILPSLQEIKKHFSSSGEFYVRAYDEKHD 216
Typhimurium SL1344              77  SACLHYNIHHRQDILSSKNTASPTVGLDSAIVDKIIFGHELNQSYCLNSIDEVEKEILNRYDIKRE-SSFIISAENYIAPIIGECRHDFNAVVICEYDKKPYVQFIDSWKTSNILPSLQEIKKHFSSSGEFYVRAYDEKHD 216
Typhimurium 4/74                77  SACLHYNIHHRQDILSSKNTASPTVGLDSAIVDKIIFGHELNQSYCLNSIDEVEKEILNRYDIKRE-SSFIISAENYIAPIIGECRHDFNAVVICEYDKKPYVQFIDSWKTSNILPSLQEIKKHFSSSGEFYVRAYDEKHD 216
Typhimurium DT2                 77  SACLHYNIHHRQDILSSKNTASPTVGLDSAIVDKIIFGHELNQSYCLNSIDEVEKEILNRYDIKRE-SSFIISAENYIAPIIGECRHDFNAVVICEYDKKPYVQFIDSWKTSNILPSLQEIKKHFSSSGEFYVRAYDEKHD 216
Typhimurium LT2                 77  SACLHYNIHHRQDILSSKNTASPTVGLDSAIVDKIIFGHELNQSYCLNSIDEVEKEILNRYDIKRE-SSFIISAENYIAPIIGECRHDFNAVVICEYDKKPYVQFIDSWKTSNILPSLQEIKKHFSSSGEFYVRAYDEKHD 216
Dublin                          77  SACLHYNIHHRQDILSSKNTASPTVGLDSAIVDKIIFGHELNQSYCLNSIDEVEKEILNRYDIKRE-SSFIISAENYIAPIIGECRHDFNAVVICEYDKKPYVQFIDSWKTSNILPSLQEIKKHFSSSGEFYVRAYDEKHD 216
Choleraesuis RF-1               77  SACLHYNIHHRQDILSSKNTASPTVGLDSAIVDKIIFGHELNQSYCLNSIEEVEKEILNRYDIKRE-SSFIISAENYIAPIIGECRHDFNAVVICEYDKKPYVQFIDSWKTSNILPSLQEIKKHFSSSGEFYVRAYDEKHD 216
Choleraesuis OU7025             77  SACLHYNIHHRQDILSSKNTASPTVGLDSTIVDKIIFGHELNQSYCLNSIDEVEKEILNRYDIKRE-SSFIISAENYIAPIIGECRHDFNAVVICEYDKKPYVQFIDSWKTSNILPSLQEIKKHFSSSGEFYVRAYDEKHD 216
Pullorum                        77  SACLHYNIHHRQDILSSKNTASPTVGLDSAIVDKIIFGHELNQSYCLNSIDEVEKEILNRYDIKRE-SSFIISAENYIAPIIGECRHDFNAVVICEYDKKPYVQFIDSWKTSNILPSLQEIKKHFSSSGEFYVRAYDEKHD 216
Gallinarum 1910/93              77  SACLHYNIHHRQDILSSKNTASPTVGLDSAIVDKIIFGHELNQSYCLNSIDEVEKEILNRYDIKRE-SSFIISAENYIAPIIGECRHDFNAVVICEYDKKPYVQFIDSWKTSNILPSLQEIKKHFSSSGEFYVRPYDEKHD 216
Gallinarum 72/805               77  SACLHYNIHHRQDILSSKNTASPTVGLDSAIVDKIIFGHELNQSYCLNSIDEVEKEILNRYDIKRE-SSFIISAENYIAPIIGECRHDFNAVVICEYDKKPYVQFIDSWKTSNILPSLQEIKKHFSSSGEFYVRAYDEKHD 216
Typhimurium ST313               77  SACLHYNIHHRQDILSSKNTASPTVGLDSAIVDKIIFGHELNQSYCLNSIDEVEKEILNRYDIKRE-SSFIISAENYIAPIIGECRHDFNAVVICEYDKKPYVQFIDSWKTSNILPSLQEIKKTLLIIRGILCQGL----- 211
```

Figure S1. Amino acid alignment of SpvD sequences.

The alignment was prepared using BioEdit software (http://www.mbio.ncsu.edu/bioedit/bioedit.html). The protein BLAST search was performed with BLASTP 2.2.29+ software (BLOSUM62 matrix; E threshold= 1.0) using *S*. Typhimurium 14028s SpvD input sequence (http://www.uniprot.org/uniprot/A0A0F6AW50). Sequences lacking a significant portion of the protein or the catalytic triad components were omitted. Polymorphisms are highlighted in yellow and catalytic triad positions are marked with red asterisks.

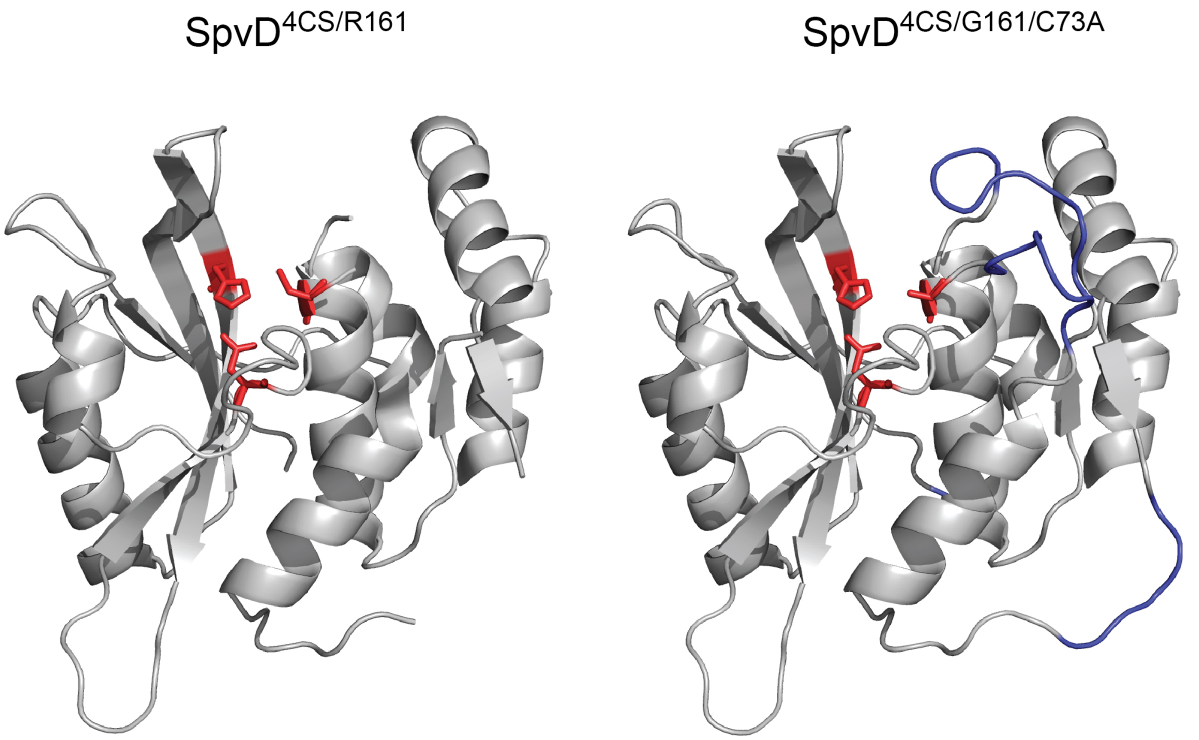

Figure S2. Comparison of $SpvD^{4CS/A154/R161}$ and $SpvD^{4CS/A154/G161/C73A}$ structures. Catalytic triad residues are represented with red sticks. Additional loop regions that were mapped in $SpvD^{4CS/A154/G161/C73A}$ structure (right) are coloured in blue.
